# Performance and Emission Characteristics of a Small Gas Turbine Engine Using Hexanol as a Biomass-Derived Fuel

**DOI:** 10.3390/ma17236011

**Published:** 2024-12-09

**Authors:** Tomasz Suchocki

**Affiliations:** Centre of Heat and Power Engineering, Institute of Fluid Flow Machinery, Polish Academy of Sciences, 80-231 Gdańsk, Poland; tsuchocki@imp.gda.pl; Tel.: +48-585-225-337

**Keywords:** hexanol, biomass-derived fuel, gas turbine engine, emission characteristics, renewable fuels, bioalcohols, TSFC, alternative aviation fuels, lignocellulosic materials

## Abstract

The global transition to renewable energy has amplified the need for sustainable aviation fuels. This study investigates hexanol, a biomass-derived alcohol, as an alternative fuel for small-scale gas turbines. Experimental trials were conducted on a JETPOL GTM-160 turbine, assessing blends of 25% (He25) and 50% (He50) hexanol with kerosene (JET A) under rotational velocities ranging from 40,000 to 110,000 RPM. The parameters measured included thrust-specific fuel consumption (TSFC), turbine inlet and outlet velocities, and the emission indices of NO_x_ and CO. The results demonstrated that the He25 and He50 blends achieved comparable thermal efficiency to pure JET A at high rotational velocities, despite requiring higher fuel flows due to hexanol’s lower heating value. CO emissions decreased significantly at higher velocities, reflecting improved combustion efficiency with hexanol blends, while NO_x_ emissions exhibited a slight increase, attributed to the oxygen content of the fuel. This study contributes a novel analysis of hexanol-kerosene blends in gas turbines, offering insights into their operational and emission characteristics. These findings underscore hexanol’s potential as an environmentally friendly alternative fuel, aligning with global efforts to reduce fossil fuel dependency and carbon emissions.

## 1. Introduction

Global efforts are underway to reduce long-term CO_2_ emissions, led by environmental groups and developed countries. These initiatives push for energy alternatives that are both cost-effective and environmentally friendly. Developing such energy sources aims to not only cut greenhouse gases but also to help countries lessen their reliance on oil [1]. Renewable fuels, especially those suitable for large-scale production, are a key part of this solution [2]. Among these, bioalcohols have shown particular promise due to their carbon-neutral life cycle and potential to replace fossil fuels in diesel engines and gas turbines [3,4,5].

Bioalcohols, made mainly of alkyl and hydroxyl groups, are cleaner alternatives to traditional petroleum fuels [6]. For decades, researchers have studied short-chain alcohols like methanol (one carbon atom) and ethanol (two carbon atoms), focusing on their performance and emissions in diesel engines and gas turbines [7,8]. However, using methanol and ethanol in diesel engines poses problems. They have a low cetane number, a high heat of vaporization, and they resist autoignition. Additionally, their lower energy content, poor mixing with diesel, and insufficient lubrication properties limit their practical use in diesel engines [9,10].

Recently, attention has shifted to higher alcohols (with four or more carbon atoms) because of their better physical and thermodynamic properties. Longer carbon chains improve ignition behavior and make the alcohols more hydrophobic, meaning they absorb less water, especially in cold conditions [11,12]. Compared to shorter-chain alcohols, higher alcohols have higher energy content, better ignition properties, lower heat of vaporization, improved dispersion, greater volatility, and lower risk of corrosion.

Hexanol is a six-carbon alcohol (CH_3_(CH_2_)_5_OH) with a molecular weight of 102.177 g/mol. It is gaining interest as a biofuel option. While it is only slightly soluble in water, hexanol mixes well with diesel and vegetable oils, forming stable blends without the need for emulsifiers [13,14]. This makes hexanol a strong candidate as a diesel additive [15,16,17]. However, using hexanol directly in standard diesel engines without modifications is not recommended due to potential compatibility issues [18].

Besides its potential as a fuel, hexanol is used in many industries. It is a component of adhesives, lubricants, fragrances, dyes, plasticizers, mold-release agents, solvents, personal care products, agricultural chemicals, pesticides, textiles, and leather goods. Hexanol has eight isomeric forms, with the main ones being 1-hexanol (also known as hexyl alcohol, n-hexanol, or hexan-1-ol), 2-hexanol, and 3-hexanol [19].

Compared to shorter-chain alcohols, hexanol absorbs less water and is less corrosive to engine fuel systems. Its properties—like higher viscosity, cetane number, boiling point, and energy density—help improve fuel economy [18,19,20]. Hexanol’s higher energy density means it provides more energy per volume, enhancing fuel efficiency. Its higher flash point also makes it safer to store and transport. Other properties, such as its heat of vaporization, density, autoignition temperature, viscosity, and vapor pressure, are closer to those of diesel fuel. This similarity helps reduce problems like cavitation and vapor lock in fuel systems [21].

Hexanol is seen as a promising future fuel for both modified and unmodified diesel engines. It can be added to diesel and biodiesel blends to improve combustion and fuel properties [22,23]. Its characteristics are similar to diesel, and it mixes well with petroleum-based fuels [24]. Under low intake pressures, hexanol is even more suitable for diesel engines than butanol. It is also being considered as an aviation fuel and could be used as jet fuel after certain chemical processes. Its higher viscosity makes it a potential substitute for aviation kerosene.

To reduce greenhouse gas emissions from diesel engines, fuels that ignite easily at low temperatures are preferred. Hexanol meets this need by offering efficient combustion and lower emissions [25]. It can also stabilize diesel micro-emulsions, improving fuel characteristics and engine performance. Therefore, hexanol has strong potential as a future alternative fuel for diesel engines, and it could replace conventional transportation fuels.

Despite these promising properties, there is limited research on using hexanol in gas turbines. While higher alcohols like pentanol have been tested in diesel engines, hexanol’s use in gas turbines has not been widely studied. Because testing gas turbines is expensive and complex, small turbines—like those used in drones or model aircraft—offer a practical alternative for research [26,27,28].

The aim of this study is to investigate the performance of a small gas turbine when operating on hexanol-based fuel. Factors such as static thrust, fuel consumption, thrust-specific fuel use, turbine inlet and outlet temperatures, as well as NO*_x_* and CO emissions at varying loads, will be analyzed. This research is intended to assess the potential advantages of using hexanol as a renewable fuel in gas turbines, contributing to efforts aimed at reducing greenhouse gas emissions and reliance on fossil fuels.

## 2. Production Methods of Hexanol

### 2.1. Renewable Production Methods

In renewable pathways, hexanol is generated through the fermentation of lignocellulosic biomass and agricultural residues like rice straw, corn stalks, and wood waste. Microorganisms such as *Clostridium kluyveri* and engineered strains of *Escherichia coli* play a crucial role in converting sugars into hexanol. The process begins with the preparation and pretreatment of biomass to break down complex structures and enhance the availability of cellulose. This is achieved through mechanical milling, washing, or chemical hydrolysis [25,29,30,31,32,33]. The efficiency of alcohol production heavily depends on pretreatment methods that enhance sugar release and enzymatic hydrolysis. Advanced methods like acid hydrolysis, enzymatic saccharification, or steam explosion can achieve sugar recovery rates of 60–90%, significantly boosting fermentation yields. Two-step approaches, such as nitrogen explosive decompression, can optimize hemicellulose recovery and reduce inhibitors, achieving up to 80% theoretical yields for specific biomasses like barley straw. Challenges like fermentation inhibitors (e.g., furfural, organic acids) can be mitigated by detoxification or using robust microorganisms (e.g., *Saccharomyces cerevisiae*, *Zymomonas mobilis*) [34,35,36,37].

Fermentation follows. The accessible sugars are transformed into hexanol along with other alcohols and by-products. The resulting mixture is then subjected to separation and purification techniques like centrifugation, filtration, and extractive distillation to isolate hexanol at the desired purity levels. Catalytic upgrading processes may also be employed to convert fermentation intermediates into high-purity hexanol [38].

There is another renewable method for producing hexanol. It involves synthesizing hexanol from syngas components—carbon monoxide, hydrogen, and carbon dioxide. These gases come from industrial waste gases. In the fermentation process, acetogenic bacteria are used. These bacteria convert the gases into hexanol through a series of biochemical reactions. Initially, waste gases are purified to remove contaminants such as sulfur compounds and nitrogen oxides. The purified gases are then fed into bioreactors where bacteria like *Clostridium carboxidivorans* facilitate the conversion. The fermentation process typically proceeds through two stages: acidogenesis, producing acids like acetate and butyrate, and solventogenesis, where these acids are converted into alcohols, including hexanol. The final product is extracted and purified from unreacted gases and other by-products using methods such as distillation [25].

### 2.2. Non-Renewable Production Methods

Hexanol can also be synthesized from petroleum resources through a series of chemical processes. The production begins by selecting suitable hydrocarbons obtained from crude oil refining. These hydrocarbons undergo cracking, a process in which they are heated to high temperatures in the presence of steam. This causes large hydrocarbon molecules to break down into smaller ones like ethylene and propylene [39,40].

These smaller olefins then participate in oligomerization reactions to form longer-chain olefins such as hexenes. Catalysts like zeolites or supported metals facilitate this process under specific temperature and pressure conditions. The hexenes produced are then subjected to hydroformylation. In this step, they react with carbon monoxide and hydrogen in the presence of catalysts—usually rhodium or cobalt complexes—to form aldehydes [41].

Subsequently, the aldehydes are hydrogenated to produce hexanol. This step involves adding hydrogen over catalysts like nickel or palladium at elevated temperatures and pressures. The crude hexanol obtained is then purified through distillation or other separation techniques to remove impurities and achieve the quality required for industrial applications [41].

## 3. The Use of Hexanol as a Fuel in Combustion Engine

### 3.1. Application of Hexanol in Diesel Engines

Based on the review article by Yahya Çelebi [42], various studies have investigated the use of hexanol as a fuel additive in diesel engines. The research primarily focuses on three types of blends: hexanol with diesel fuel, hexanol with biodiesel, and hexanol in combination with both diesel and biodiesel. Each type of blend offers unique benefits and challenges in terms of engine performance, combustion characteristics, and exhaust emissions.

Numerous studies have explored the integration of hexanol into biodiesel to enhance diesel engine performance and emission profiles [16,20,43,44,45,46]. Researchers have found that hexanol mixes readily with biodiesel and can be utilized in compression ignition engines without hardware modifications, making it a practical and versatile additive [13,17]. The inclusion of hexanol generally results in reduced cylinder pressure and heat release rates due to its lower cetane number, which extends the ignition delay period [17,34]. This extension can lower peak combustion temperatures and pressures, potentially mitigating the risk of engine knocking [34,35,36].

The addition of hexanol typically leads to significant decreases in emissions of carbon monoxide (CO) and hydrocarbons (HC) [13,17,36]. This improvement is attributed to the extra oxygen content and reduced viscosity of the hexanol-biodiesel blend, which enhance fuel evaporation and promote more complete combustion [35,37]. Emissions of smoke, soot, and particulate matter (SSP) also tend to decrease, as the oxygen present in hexanol aids in oxidizing soot particles [17,34,36]. However, the impact on nitrogen oxide (NO_x_) emissions is inconsistent across different studies. Some report increases in NO_x_ due to higher in-cylinder temperatures from the abundant oxygen content, especially at higher hexanol concentrations and engine loads [13,36]. Others observe decreases in NO_x_ emissions [17,34].

Regarding engine efficiency, the incorporation of hexanol often results in higher brake thermal efficiency (BTE) [17,34], while brake-specific fuel consumption (BSFC) may show mixed results [13,35]. The lower energy density of hexanol can lead to increased BSFC, although more complete combustion can improve BTE [36,37].

Extensive research has been conducted on blending hexanol with diesel fuel to improve engine performance and reduce reliance on fossil fuels [14,15,20,21,38,39,40,41,42,43]. Studies indicate that up to 40% hexanol can be added to diesel without significant modifications to the engine [14,15,38]. Higher percentages of hexanol typically lead to increased cylinder pressures and heat release rates, particularly under low-load conditions [15,20]. This effect is due to hexanol’s lower cetane number and calorific value, causing a longer ignition delay and allowing more fuel to accumulate before combustion [14,21,39].

The influence of hexanol on emissions is varied. CO emissions often increase due to incomplete combustion from prolonged ignition delays [20,40], while HC emissions show mixed trends [15,41]. NO*_x_* emissions also display variable behavior; some studies report increases [14,42], while others note decreases [15,20,43]. Generally, NO*_x_* emissions tend to decrease as the proportion of hexanol in the blend increases, especially at high engine loads, possibly due to the cooling effect from hexanol’s high latent heat of vaporization [14,15,20]. SSP emissions consistently decrease with higher hexanol content, as the oxygen in hexanol promotes the oxidation of soot particles [14,15,38].

From a performance standpoint, BSFC usually increases with higher hexanol content because of its lower heating value, and BTE may decrease [15,21]. The use of exhaust gas recirculation (EGR) with hexanol–diesel blends extends the ignition delay due to inert gas dilution, reducing the adiabatic flame temperature and resulting in significant reductions in NO*_x_* and SSP emissions [14,15,39]. In dual-fuel combustion modes, CO and HC emissions may increase with hexanol use, while NO_x_ and SSP emissions decrease [39,41]. Research on ternary blends of hexanol, biodiesel, and diesel fuel has demonstrated their potential as alternative fuel solutions for diesel engines [10,44,45,46,47,48,49,50,51,52]. Experimental studies have confirmed that these mixtures can be successfully utilized in conventional diesel engines without hardware modifications, with hexanol content typically ranging from 5% to 15% by volume [10,44,45]. However, they may exhibit more complex combustion behaviors [46,47].

Adding hexanol to biodiesel-diesel blends generally reduces cylinder pressure and heat release rates [48,49]. This is due to the cooling effect of hexanol and the increased specific heat capacity of the blend [50,51]. The blend absorbs more heat during evaporation, lowering in-cylinder temperatures [10,44,46]. The presence of hexanol in these ternary blends often results in reduced exhaust gas temperatures and shorter ignition delays, which are attributed to the higher cetane number of the mixture, facilitating better evaporation and combustion [44,47].

Emissions of CO, HC, and SSP (Smoke, Soot, and Particulate Matter) consistently decrease as the hexanol percentage increases, owing to the additional oxygen promoting more complete combustion and enhanced conversion of CO to CO_2_ [45,48,52]. The impact on NO*_x_* emissions varies, with some studies indicating increases [46,49] and others decreases [44,50]. However, NO*_x_* levels generally diminish with increasing engine speed [10,47]. Higher hexanol content may lead to decreased BTE and increased BSFC, reflecting the need to balance fuel economy and performance due to the lower heating value of hexanol and biodiesel [48,51].

Preheating the ternary blend has been shown to significantly reduce BSFC, ignition delay, CO, HC, and SSP emissions while improving BTE, heat release rates, and cylinder pressures [44,50]. Employing EGR is effective in reducing NO*_x_* emissions when using ternary blends, with an optimal EGR rate around 10% [14,15,48]. Additionally, hexanol can serve as an ignition improver in ternary blends at concentrations of up to 1% [52].

### 3.2. Literature Review on Alcohols in Gas Turbines

Based on the comprehensive analysis of higher alcohols as alternative fuels in gas turbine engines, several consistent patterns emerge across multiple studies (Table 1). Research conducted by Mendez et al. [47,48] and Chen et al. [49] predominantly showed increases in thrust-specific fuel consumption (TSFC) when testing various alcohols, while the temperatures TIT (turbine inlet temperature), TOT (turbine outlet temperature), and EGT (exhaust gas temperature) typically remained constant or increased. In terms of emissions, most studies, including those by Buffi et al. [50] and Suchocki et al. [3], demonstrated reductions in both CO and NO emissions. However, the current study on hexanol exhibits distinctive characteristics that set it apart from previous research. Unlike earlier findings, this hexanol investigation maintains stable TSFC values while achieving lower operational temperatures. Most notably, it presents a unique emission profile where NO emissions increase, contrasting with the decreasing trends observed in all previous studies, including those with pentanol. This research, conducted on the engine JETPOL GTM160 (Poznań, Poland), suggests that as the carbon chain length of alcohol fuels increases, new and unexpected emission characteristics emerge. This study’s findings, particularly the stable fuel consumption coupled with increased NO emissions, represent a significant deviation from the established patterns in alcohol-based fuel research. This distinction becomes especially apparent when compared to studies of lower alcohols, indicating that the relationship between alcohol carbon chain length and engine performance/emissions may be more complex than previously understood in gas turbine applications.

### 3.3. Motivation

Despite the extensive research on hexanol blends in diesel engines, there is a significant research gap regarding their application in gas turbine engines. Gas turbines are crucial in the power generation and aviation sectors, and exploring the use of hexanol in these engines could lead to improvements in combustion efficiency and emissions reductions similar to those observed in diesel engines. The oxygenated nature of hexanol may enhance flame stability and reduce soot formation in turbine combustion chambers. Investigating hexanol’s potential in gas turbines could provide valuable insights and contribute to the development of cleaner and more efficient energy systems.

A secondary aim of this study is to conduct a preliminary evaluation of alcohols like hexanol for blending with plastic-derived fuels, which, despite favorable energy properties, are characterized by high CO and NO*_x_* emissions [54,55,56]. Assessing the compatibility of alcohol blends with these plastic-based fuels could open pathways to mitigating emissions while harnessing the energy potential of waste-derived sources.

## 4. Test Stand and Methodological Approach

### 4.1. Experimental Facility and Exhaust Gas Composition Measurement Measurements

**Figure 1:**
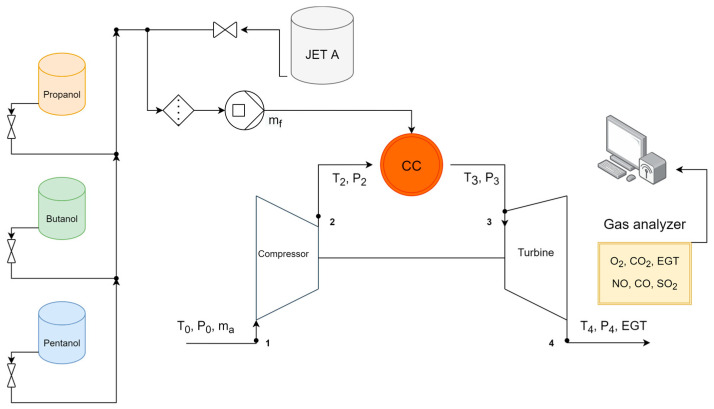
Schematic diagram of a miniature gas turbine engine experimental facility and measurements [3].

Conducting measurements and experimental studies on stationary gas turbines is often expensive and complex. To reduce research costs, a miniature gas turbine engine was employed for laboratory-scale experiments. [57,58,59,60,61,62]. The studies detailed in this paper were carried out using a JETPOL GTM-160 gas turbine engine at the Institute of Fluid-Flow Machinery, Polish Academy of Sciences in Gdańsk. A schematic representation of the experimental setup is illustrated in Figure 1.

Technical performance data of the gas turbine engine are shown in Table 2, highlighting key parameters such as maximum thrust, fuel consumption rate, and rotational speed. These parameters provide an in-depth understanding of the engine’s capabilities and operational limits, supporting the analysis of performance under various experimental conditions.

Prior research on alternative fuels utilizing miniature jet engines has been undertaken [55,63,64,65,66]. The previous studies primarily focused on the technologies used in the production of synthetic hydrocarbons approved for use in aviation turbine engines. The technical performance data of the micro gas turbine are presented in Table 3. Detailed measurements are compiled in Table 4. The GTM-160 engine comprises a single-stage centrifugal compressor and a single-stage axial turbine mounted on the same shaft, along with a reverse-flow annular combustor featuring six evaporators. Upon measuring the exhaust emission concentrations, the emission index for each harmful exhaust gas component were calculated. The emission index for a component (i) is defined as the ratio of the mass of that component to the mass unit of fuel consumed during combustion as Equation (1) [67]:

(1) $${EI}_{i}=\frac{m_{i,emitted}}{m_{f,burned}}$$

The emission index serves as a valuable parameter that clearly quantifies the amount of a pollutant produced per unit mass of fuel, independent of the dilution processes of combustion products and the actual efficiency of the combustion process. In the combustion of hydrocarbon fuels with air, the emission index can be determined based on the concentrations of specific emission components (molar fractions) and all components containing a carbon atom (C). Assuming that all carbon from the fuel is converted into CO_2_ and CO in the exhaust gases (with soot being neglected), the emission index can be expressed as Equation (2) [67]:

(2) $${EI}_{i}=\left(\frac{\chi_{i}}{\chi_{CO}+\chi_{{CO}_{2}}}\right)\left(\frac{{xMW}_{i}}{{MW}_{F}}\right)$$

### 4.2. Fuels

Table 3 and Table 4 summarize the physico-chemical properties of the alcohols and kerosene, respectively. Hexanol, when compared to shorter-chain alcohols ranging from C1 to C6, exhibits a higher density, viscosity, heat of combustion, cetane number, boiling point, and flash point. It also has a significantly lower latent heat and a reduced autoignition temperature. Its molecular composition contains less oxygen and a higher weight percentage of carbon. The fuel used in this study was purchased as 1-hexanol (CAS No. 111-27-3).

**Table 3:** Physicochemical properties of tested fuels.

	JET A1	He25 Hexanol	He50 Hexanol
Molecular formula	C_13_H_26_		
Mass Percentage of Oxygen [%]	0	3.92	7.83
Molecular Weight [kg/kmol]	182	162.0	142.1
Density at 15 °C [kg/m³]	810	812	815
Viscosity at 15 °C [cP]	3.80	4.22	4.65
Lower Heating Value [MJ/kg]	43.28	41.46	39.64
Latent Heat of Vaporization [kJ/kg]	330	355	380
Boiling Point [°C]	118	127.8	137.5

He25 mixture contains 25% hexanol and 75% wt. JET A. He50 mixture contains 50% hexanol and 50% wt. JET A.

**Table 4:** Physicochemical properties of selected alcohols (C1-C6) [3,68,69].

Property	Methanol	Ethanol	n-Butanol	n-Hexanol
Molecular Formula	CH_3_OH	C_2_H_5_OH	C_4_H_9_OH	C_6_H_1__3_OH
Molecular Structure				
Density at 15 °C (g/mL)	0.791	0.789	0.810	0.820
Kinematic Viscosity at 40 °C (mm²/s)	0.58	1.08	2.22	3.57
Lower Heating Value (MJ/kg)	20.1	26.9	33.1	36.0
Heat of Vaporization (kJ/kg)	1162	918	585	285
Cetane Number	3.8	5–8	17	25
Boiling Point (°C)	65	78	117	157
Flash Point (°C)	12	13	35	62
Autoignition Temperature (°C)	463	420	345	280
Oxygen Content (% by weight)	49.93	34.73	21.59	15.62
Carbon content (wt%)	37.49	52.15	64.82	

To evaluate the gas turbine’s performance and emissions, several parameters were measured using specialized instruments. These measurements encompassed temperatures, pressures, thrust, fuel flow, rotational speed, and exhaust gas concentrations.

○ Temperature measurements (T_1_–T_4_): Type K thermocouples (provided by Czah, Katowice, Poland) were utilized to record the temperatures at the compressor and turbine inlets and outlets. These sensors operate within a range of 0 to 1100 °C with a precision of 1 °C and an uncertainty margin of ±1 °C.
○ Pressure measurements (P_1_–P_4_): Digital pressure transducers (provided by Aplisense, Warsaw, Poland) measured the pressures at key points in the compressor and turbine. Sensor P_1_ covered a range from 0 to 0.98 bar, while sensors P_2_ to P_4_ spanned from 0 to 9.8 bar. The devices have a resolution of 0.01 bar and an uncertainty of ±1.0%.
○ Static thrust: A strain gauge-based load cell assessed the static thrust produced by the engine, capable of measuring forces between 0 and 200 N with a resolution of 1 N and an uncertainty of ±1 mV/V.
○ Fuel flow Rate: The volumetric flow rate of fuel was monitored using an oval gear flow meter, suitable for flows from 0.5 to 100 L per hour (L/h). The flow meter offers a resolution of 0.01 L/h and has an uncertainty of ±0.5%.
○ Rotational speed: Engine speed was tracked using a magnetic pickup tachometer, effective over a range of 0 to 200,000 RPM. The instrument provides readings with a resolution of 1 RPM and an uncertainty of ±0.5%.
○ Exhaust gas analysis (provided by Madur; Ga 40plus): Concentrations of oxygen, carbon monoxide, nitric oxide, and sulfur dioxide in the exhaust were measured using electrochemical gas analyzers. These devices offer high-resolution measurements and have specified absolute and relative uncertainties to ensure data accuracy.

### 4.3. Procedure

Experimental results were recorded for engine speeds ranging from 40,000 to 110,000 RPM. At each rotational speed, measurements commenced three minutes after achieving thermal stabilization of each combustion chamber sector and establishing steady-state parameters. Each mixture of JET A1 with hexanol was evaluated under the same throttling valve settings as pure JET A, maintaining consistent environmental conditions. The throttling valve was adjusted to allow the engine to reach the desired speed while using the base fuel. Subsequently, aviation kerosene was replaced with fuel blends containing 25% and 50% hexanol by volume. In the notation of fuel blends, each symbol consists of an abbreviation (He—hexanol (C_6_H_13_OH)) and a number indicating the volumetric percentage of hexanol in the mixture with JET A. For example, the He25 mixture contains 25% hexanol and 75% JET A. After testing all mixtures at a specific throttle valve setting, the procedure was repeated for the next setting (or engine speed).

The following parameters were measured and analyzed over a wide range of turbine loads: turbine inlet temperature, exhaust gas temperature (EGT), specific fuel consumption, static thrust, and the NO_x_ and CO emission indices. To minimize experimental uncertainties and enhance the reliability of the test results, engine performance and exhaust emissions tests were conducted twice for all hexanol–aviation kerosene blends.

## 5. Results and Discussion of Gas Turbine Performance Characteristics

### 5.1. Thrust-Specific Fuel Consumption and Fuel Flow

**Figure 2:**
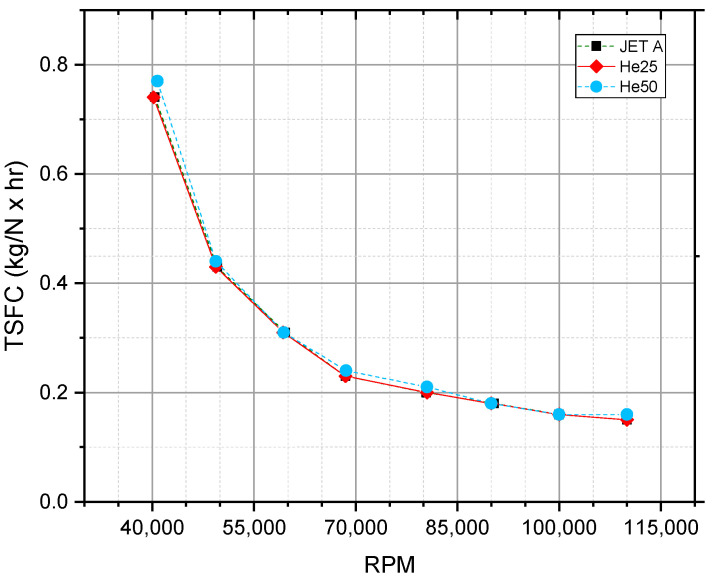
TSFC as a function of RPM for three fuel types: JET A, He25, and He50.

The TSFC, represented in Figure 2, is a measure of the fuel consumed per unit of thrust generated by the engine. This parameter is inversely related to the thermal efficiency of the fuel: a lower TSFC signifies higher thermal efficiency [53]. Figure 2 illustrates that, across all fuel compositions (JET A, He25, and He50), the TSFC decreases with increasing rotational speed, indicating improved fuel efficiency at higher rpm. For lower speeds, the TSFC is significantly higher, with more pronounced differences between fuel types at low rpm. The convergence of the TSFC values at higher rpm (above 85,000 RPM) suggests that at high speeds, the efficiency differences between fuel types diminish, likely due to the greater engine power output stabilizing the fuel consumption rates. While lower-chain alcohols like ethanol and propanol tend to increase TSFCs due to their lower energy densities [53,54], hexanol blends in our study maintained TSFC levels comparable to JET A at higher rpm. This aligns with findings for pentanol blends [1], suggesting that higher alcohols may offer better fuel efficiency in gas turbines than lower alcohols.

This trend reflects that at lower rpm, the turbine operating with He25 and He50 mixtures shows a slightly higher TSFC compared to JET A, implying a minor efficiency loss due to lower heating values or combustion properties of the alternative fuels. However, as RPM increases, the turbine’s performance with He25 and He50 aligns closely with JET A, indicating that these mixtures can achieve comparable efficiency at high loads.

**Figure 3:**
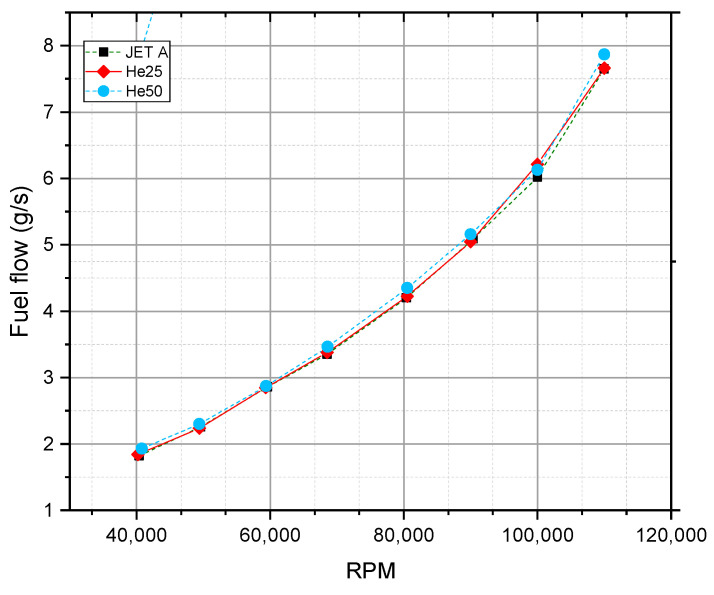
TSFC as a function of RPM for three fuel types: JET A, He25, and He50.

Figure 3 provides an analysis of fuel flow rates (g/s) as a function of turbine RPM in a micro gas turbine operating on mixtures of hexanol and JET A. The data highlights the increasing fuel flow at higher rpm due to greater energy demands at higher speeds. Notably, the He50 mixture exhibits slightly higher fuel flow compared to He25 and JET A, particularly at elevated rpm. This indicates that the energy yield per unit mass for He50 may be slightly less efficient than JET A, possibly due to the blending effects of hexanol with JET A. These variations underline the impact of hexanol’s calorific properties on combustion performance and turbine operation.

When considering the relationship between TSFC and fuel flow, it becomes evident that although alternative fuel mixtures (He25 and He50) can achieve efficiencies comparable to JET A at high speeds, they require slightly higher fuel flows. This implies a trade-off: while these mixtures are effective in maintaining low TSFC at high rpm, they may not be as efficient as JET A in terms of absolute fuel consumption due to potentially lower calorific values.

Overall, these graphs (Figure 2 and Figure 3) illustrate the performance nuances of different fuel types in gas turbine engines, highlighting the influence of fuel composition on both TSFC and fuel flow, with JET A generally showing a slight advantage in fuel efficiency at comparable speeds.

### 5.2. Turbine Inlet Temperature

**Figure 4:**
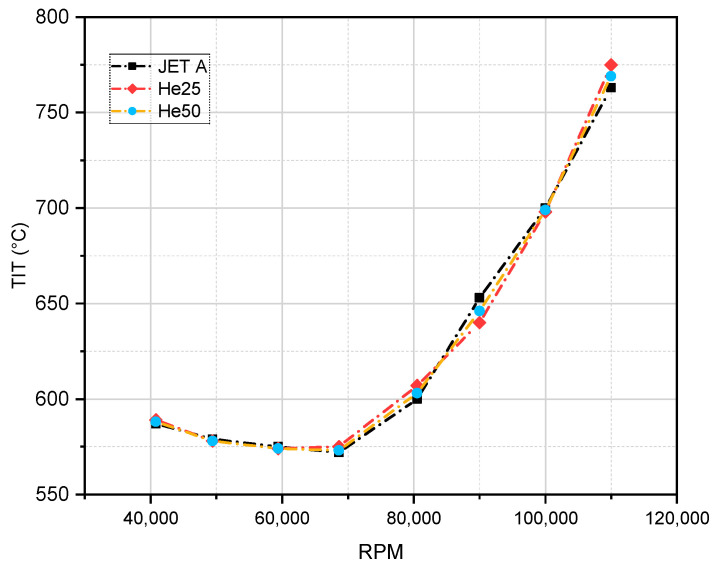
Turbine inlet temperature as a function of RPM for three fuel types: JET A, He25, and He50.

Figure 4 illustrates the variation in turbine inlet temperature (TIT) as a function of RPM for different fuel mixtures, specifically JET A, He25, and He50. TIT is a critical parameter that greatly impacts both the performance and emissions profile of a gas turbine engine. In this experiment, the TIT values were measured using an average of three thermocouples placed upstream of the turbine’s guide vane row, positioned at the midpoint of the vane height. This arrangement provides an accurate representation of the temperature entering the turbine stage, essential for evaluating fuel efficiency and emissions characteristics.

As observed in Figure 4, the turbine inlet temperature (TIT) initially decreases with increasing RPM, reaching a minimum at around 70,000 RPM. Beyond this threshold, the TIT begins to rise steadily as RPM increases, peaking at approximately 725 °C around 110,000 RPM for all fuel compositions (JET A, He25, and He50). The TIT values for He25 and He50 are very similar to those of JET A, with only minor variations observed across the RPM range. This similarity indicates that the combustion characteristics and resulting temperatures of these alternative fuel mixtures closely match those of the baseline JET A fuel.

The TIT has a critical impact on nitrogen oxides (NO*_x_*) emissions, as described by the Zeldovich mechanism, which explains that higher temperatures facilitate the breakdown of nitrogen (N_2_) and oxygen (O_2_) molecules, leading to the formation of NO*_x_*. As the TIT increases, particularly at higher rpm, NO*_x_* emissions are expected to rise due to the elevated temperatures creating favorable conditions for NO*_x_* formation. Given that the TIT exceeds 775 °C at the highest rpm, NO*_x_* emissions are likely to be higher in this range, regardless of the fuel mixture used.

While the He25 and He50 mixtures may offer a slight reduction in the TIT compared to JET A at certain rpm, the differences are minimal, suggesting that NO*_x_* emissions across all three fuel types will be quite similar, particularly at higher rpm, where the TIT values converge. Overall, Figure 4 suggests that while alternative fuels might offer a marginal benefit in NO*_x_* reduction at low to moderate rpm, this advantage diminishes at higher rpm due to the similar TIT levels across the tested fuels.

Our study showed minimal changes in TITs with hexanol blends, similar to the findings of studies on pentanol [1]. In contrast, some studies reported increases in TITs with ethanol and propanol blends [53,58]. This indicates that higher alcohols may not significantly impact the TIT, potentially aiding in the thermal management of the turbine.

### 5.3. Gas Turbine Emission Index

**Figure 5:**
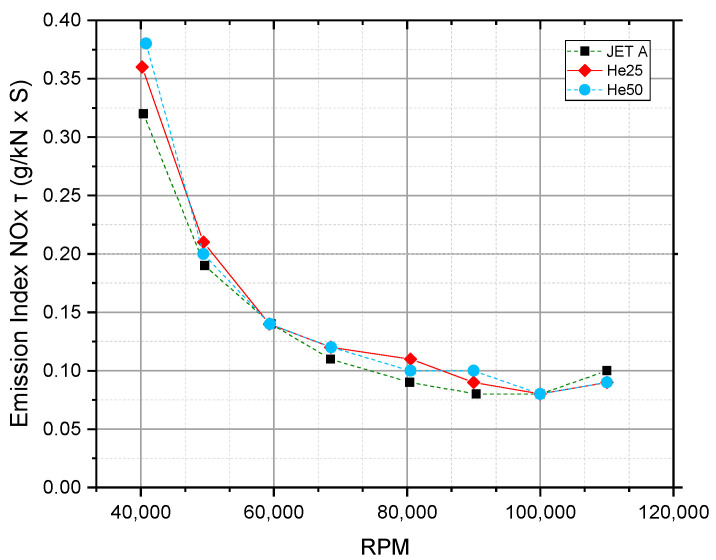
Emission Index of NO (g/kN·s) as a function of RPM for three fuel types: JET A, He25, and He50.

Figure 5 presents the Emission Index of NO*_x_* (g/kN·s) as a function of RPM for three fuel types: JET A, He25, and He50.

As RPM increases from 40,000 to around 85,000, there is a marked decrease in the NO*_x_* emission index for all tested fuels. This trend is likely associated with lower combustion temperatures at mid-range rpm, which reduce the conditions conducive to NO_X_ formation via thermal (Zeldovich) mechanisms. This mechanism, which predominates in high-temperature combustion, involves the dissociation of nitrogen (N_2_) and oxygen (O_2_) molecules, enabling the formation of NO*_x_*. As combustion efficiency stabilizes and the temperature decreases with rising RPM, NO*_x_* emissions correspondingly drop.

At rpm beyond approximately 85,000, the emission index levels off, with a minor increase observable as the RPM approaches 110,000. This stabilization suggests that the thermal NO_X_ formation rate begins to balance out with increasing engine speed, potentially due to the competing effects of higher temperatures and shorter residence times in the combustion chamber. The slight increase at very high rpm could be attributed to elevated combustion temperatures at high loads, which again favor NO_X_ production despite reduced residence time.

The NO*_x_* emission profiles of JET A, He25, and He50 remain closely aligned across the RPM spectrum, indicating that the addition of hexanol in He25 and He50 does not significantly alter NO_X_ formation under these conditions. This suggests that while He25 and He50 may have slightly lower calorific values, their combustion characteristics in terms of NO*_x_* formation remain similar to JET A. At lower rpm, He50 shows a minor increase in NO*_x_* emissions relative to JET A, possibly due to a less complete combustion process at reduced speeds, but this effect diminishes as the RPM increases, leading to the convergence of emission values.

The data suggest that NO_X_ emissions can be effectively managed by maintaining engine operation within an optimal RPM range (around 85,000), where NO*_x_* formation is minimized. However, at higher rpm, the thermal advantages of He25 and He50 over JET A appear negligible in terms of NO_X_ reduction, as all fuels converge to similar emission levels. For applications where NO_X_ emissions are a critical concern, the use of He25 or He50 may offer minor advantages at low to mid-rpm but will not significantly impact emissions at high engine loads.

The increase in NO*_x_* emissions with hexanol blends observed in our study contrasts with the reductions reported in other studies using lower-chain alcohols [53,54,58]. This suggests that the combustion of hexanol may produce higher temperatures or different reaction pathways, leading to increased NO*_x_* formation, highlighting the need for further research into the combustion mechanisms of higher alcohols.

**Figure 6:**
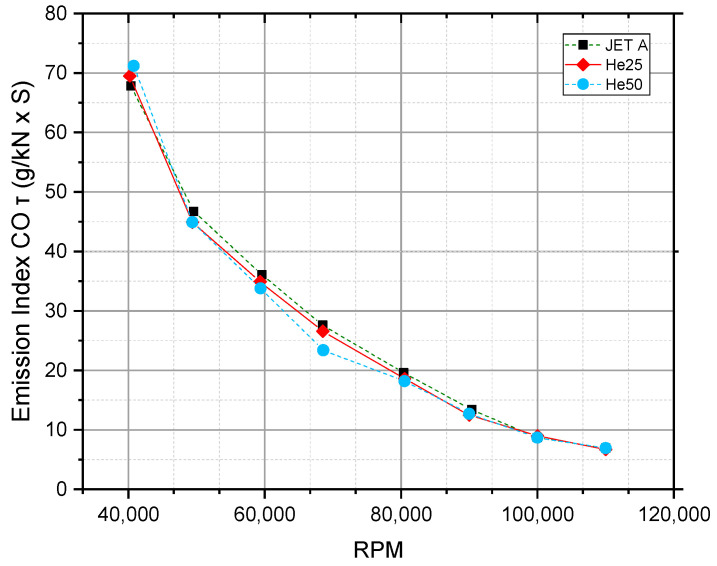
Emission index of CO (g/kN·s) as a function of RPM for three fuel types: JET A, He25, and He50.

Figure 6 displays the emission index of CO (g/kN·s) as a function of RPM for the fuel types JET A, He25, and He50.

Overall, the CO emission index shows a strong decreasing trend as RPM increases, dropping from approximately 70 g/kN·s at 40,000 RPM to below 10 g/kN·s at 110,000 RPM. This decline in CO emissions is indicative of more complete combustion at higher engine speeds, where increased air and fuel mixing, along with higher combustion temperatures, facilitate the oxidation of CO into CO_2_.

For all three fuels, the emission trends are quite similar, with minimal variation across the RPM range, suggesting that the addition of hexanol in He25 and He50 has a negligible effect on CO emissions under these conditions. The CO emission levels converge closely as RPM rises, with only slight deviations at lower rpm, where He50 shows a minor advantage in reducing CO emissions compared to JET A and He25.

Consistent with other studies [1,53,54,58], CO emissions decreased with the use of hexanol blends, indicating more complete combustion. This reinforces the potential of higher alcohols to improve combustion efficiency and reduce CO emissions in gas turbines.

In summary, Figure 6 indicates that CO emissions decrease significantly with increasing RPM for all fuels tested, and while He25 and He50 show minor variations in CO emissions, they do not offer substantial advantages over JET A, especially at high rpm, where CO emissions are already minimized.

## 6. Conclusions

This study presents a detailed evaluation of hexanol, a biomass-derived alcohol fuel, blended with aviation kerosene (JET A) for use in small gas turbines, specifically the JETPOL GTM-160 engine. Hexanol’s higher carbon chain length provides improved energy density, better water resistance, and compatibility with kerosene, making it an appealing candidate for applications in turbines and other combustion engines. Key parameters such as static thrust, thrust-specific fuel consumption (TSFC), turbine inlet temperature (TIT), exhaust gas temperature (EGT), and emissions of nitrogen oxides (NO*_x_*) and carbon monoxide (CO) were analyzed across a range of rotational speeds (40,000–110,000 RPM) to assess the feasibility and efficiency of hexanol as an alternative fuel.

The results indicate that while hexanol blends require slightly higher fuel flow rates than pure JET A—attributed to hexanol’s lower heating value—they provide comparable fuel efficiency at higher engine loads, particularly at speeds exceeding 85,000 RPM. Emission trends were also favorable. Mid-range rpm saw reductions in NO*_x_* emissions due to lower combustion temperatures, though this benefit diminished at peak speeds. CO emissions decreased consistently as RPM increased, suggesting more complete combustion with hexanol blends, particularly at high engine speeds.

Hexanol is a very promising alternative fuel for Poland and the European Union. It offers significant benefits over butanol, such as higher energy content, better combustion stability, lower tendency to mix with water, and greater compatibility with existing fossil fuel systems. Producing hexanol from plant-based materials like wheat straw and forestry leftovers aligns well with the EU’s Renewable Energy Directive and suits the regional climate, which favors these types of crops.

Because of its excellent properties, hexanol is especially suitable for use in aviation and industrial gas turbines, supporting the EU’s goals for renewable and advanced biofuels. This research provides valuable insights into the potential of hexanol as a renewable fuel source for gas turbines. However, more studies are essential to optimize engine parameters for increased fuel efficiency and reduced emissions.

Future investigations should explore higher hexanol concentrations, additional fuel blend configurations, and their effects on long-term engine performance, durability, and emission profiles. Expanding research to include different engine types and configurations—such as diesel and dual-fuel engines—would also enhance our understanding of hexanol’s versatility and operational benefits. In diesel engines, higher alcohols like hexanol offer improved combustion characteristics, lower particulate emissions, and potential compatibility with current engine designs with minimal modifications.

Moreover, the future of biomass-derived fuels, especially alcohols like hexanol, holds promise across various sectors of power generation and transportation. In gas turbines, alcohol-based fuels like hexanol could help reduce carbon footprints and dependency on fossil fuels. In combined heat and power (CHP) systems, biomass-derived alcohols can support more efficient, low-emission energy production for both electricity and heating. This provides a sustainable solution for industries and municipalities aiming for carbon-neutral operations.

Advancing these technologies will position hexanol as a key part of the EU’s shift toward cleaner energy, utilizing local biomass resources while cutting down greenhouse gas emissions. By integrating hexanol into various energy systems, we can leverage its benefits to support the EU’s renewable energy goals and contribute to a more sustainable future.

The lack of access to a broader range of alternative fuels limited the scope of our comparative analysis. In future studies, it would be beneficial to include a greater variety of biofuels to better assess their relative advantages and potential applications.

While promising, the production methods of hexanol remain energy-intensive and require optimization for large-scale applications. Further research is necessary to refine these processes and explore alternative raw materials, which will allow for increased efficiency and cost-effectiveness in production.

With ongoing advancements in biomass processing and fuel technology, alcohols like hexanol are poised to play a significant role in the energy sector. They offer a viable pathway toward decarbonization, improved air quality, and energy security by diversifying fuel sources and enhancing compatibility with the existing power generation infrastructure. Further large-scale studies and industrial trials will be necessary to realize the full potential of alcohol-based biomass fuels in power generation, transportation, and CHP applications, establishing a foundation for a more sustainable energy future.

## Figures and Tables

**Table 1:** The analysis of higher alcohols as alternative fuels in gas turbine engines.

Reference, Year	Tested Fuel	Test Setup	Conclusion Higher Alcohols Fraction Led to:	
			Performance	Emission
Mendez et al. [48] 2012	Ethanol (E25-E75)		Thrust ↑ TSFC ↑ TIT/TOT/EGT =	${EI}_{CO}$ ↓ ${EI}_{NO}$ ↓
Sallevelt et al. [51] 2014	Ethanol	OPRA’s 2 MWe class OP 16 gas turbine combustor Range 29–54 KW	Fully combusted Power ↑	CO = NO ↓
Buffi et al. [52] 2018	Liquefied Wood + Denatured Ethanol (LW/EtOH 75/25; 50/50 wt%)	GTP 30–67	TIT/TOT/EGT ↑	${EI}_{CO}$ ↓ ${EI}_{NO}$ ↓ ${EI}_{UHC}$ ↑
Chen et al. [49] 2017	Butyl Butyrate/Ethanol (ethanol fraction 0–50%)	Combustion chamber is fabricated based on an aero-engine	TSFC ↑ TIT/TOT/EGT =	${EI}_{CO}$ ↓ ${EI}_{NO}$ ↓ ${EI}_{UHC}$ ↑
Buffi et al. [50] 2018	Pyrolysis Oil/Ethanol (PO/EtOH 20/80%; 50/50%)	GTP 30–67	TSFC ↑ TIT/TOT/EGT =	${EI}_{CO}$ ↓ ${EI}_{NO}$ ↓
Mendez et al. [47] 2011	Propanol (P25-P100)	Small-scale gas turbine 178 N	TSFC ↑ TIT/TOT/EGT ↑	${EI}_{CO}$ ↓ ${EI}_{NO}$ ↓
Mendez et al. [53] 2014	Butanol (B50-B100)		TSFC ↑ TIT/TOT/EGT =	${EI}_{CO}$ ↓ ${EI}_{NO}$ ↓
Suchocki et al. [3] 2023	Pentanol (Pe25-Pe100)	JETPOL GTM140 140 N	TSFC ↓ TIT/TOT/EGT ↓	${EI}_{CO}$ ↓ ${EI}_{NO}$ ↓
This work 2024	Hexanol (He25-He50)	JETPOL GTM160 180 N	TSFC = TIT/TOT/EGT ↓	${EI}_{CO}$ ↓ ${EI}_{NO}$ ↑

↑: Increased. ↓: Decreased. =: No significant change.

**Table 2:** Technical performance data of the JETPOL GTM-160 gas turbine engine.

Parameter	Specification
Design Maximum Thrust	160 N, up to 170 N
Air Mass Flow Rate	0.4 kg/s
Compression Ratio	3:1
Specific Fuel Consumption	7.0 g/s (Experimental: 400 g/min at max thrust)
Compressor	Single-stage centrifugal flow (radial outflow)
Turbine	Single-stage axial flow
Maximum RPM	120,000 RPM
Minimum RPM	33,000 RPM (Experimental)
Max Turbine Inlet Temp (TIT)	700 °C
Max Exhaust Gas Temp (EGT)	550 °C
Lubrication	3–5% Aeroshell in fuel (JET A)

## Data Availability

The original contributions presented in this study are included in the article. Further inquiries can be directed to the corresponding author.
